# Welding of thin stainless-steel sheets using a QCW green laser source

**DOI:** 10.1038/s41598-024-54305-4

**Published:** 2024-02-19

**Authors:** E. Haddad, F. Poggenburg, A. Häusler, A. Olowinsky

**Affiliations:** 1 Fraunhofer Institute for Laser Technology ILT, Aachen, Germany; 2 Chair of Laser Technology LLT, Aachen, Germany

**Keywords:** Hydrogen, Fuel cells, Bipolar plates, Laser welding, Stainless steel, Green laser, Mechanical engineering, Electrical and electronic engineering, Fuel cells, Hydrogen energy, Fibre lasers

## Abstract

Bipolar plates are structured thin metal sheets and are, next to the membrane electrode assembly (MEA), one of the main components of polymer electrolyte membrane fuel cells. One of the production steps of such bipolar plates is the joining process of its two halves. Laser welding is a suitable method for such an application since it is fast, non-contact, automatable, and scalable. Particularly important aspects of the weld seam are the weld seam width and depth. In this paper, welding of stainless-steel material analogous to materials used in bipolar plates is examined. For this purpose, a newly developed quasi continuous wave (QCW) green laser source with higher beam quality is employed to assess the effect of the wavelength and the spot diameter on the welding of stainless-steel material. By using various focusing lens, different sized beam diameters below 20 µm are achieved and their influence on the final welding result—specifically concerning the seam width—are analyzed. With welding speeds starting at 500 mm/s, reduced weld seam widths (≤ 100 µm) are realized, particularly with a focusing lens of 200 mm focal distance. The suitability of such a process for thin channels of under 75 µm width is examined.

## Introduction

With the climate change crisis and the inevitable exhaustion of fossil fuel reserves, the world is in search for sustainable solutions for the near future, where the energy supply is environmentally friendly, reliable, and affordable. Ambitious goals of gradually limiting greenhouse gases (GHG) to achieve 55% reduction by 2030 compared to 1990 and net-zero by 2050 are parts of the European Union’s fit for 55 Package. This can only be accomplished by applying policies and instruments across various sectors: energy, transport, and land use for example. The use of alternative drives in the increasingly growing transport sector—a sector responsible for 27% of total EU GHG emissions in 2017—can significantly contribute to the success of such goals^1^. Alternative methods such as the fuel cell are to be used to convert renewable resources directly and efficiently such as hydrogen, which can be created in the event of an energy surplus and stored in tanks. In this regard, fuel cell technology is frequently seen as the core technology of the energy transition in its numerous fields of application^2^. Fuel cell technology significantly boosts the appeal of environmentally friendly mobility, thanks to comparably quick refueling times and the significantly higher ranges compared to battery electric vehicles (BEV). The fuel cell offers a solution to the mobility dilemma of the future, one that may demonstrate an excellent environmental balance due to the use of largely recyclable components^3^. However, a large-scale deployment and widespread market penetration of this technology are unlikely given the high production costs and poor production capabilities of a cell's primary components with the existing manufacturing techniques.

The bipolar plate is the heart of the hydrogen fuel cell and one of its main components next to the membrane electrode assembly (MEA)^4^. Various approaches exist for manufacturing bipolar plates, with the approach of a metallic bipolar plate offering the greatest advantages in terms of weight, volume, and serial production. This type of bipolar plate consists of two thin stainless-steel sheets, into which gas flow profiles are embossed, and that need to be hermetically sealed. The geometry of the gas flow channels in the flow field impacts the performance of the cell^5^. Compared to other joining processes, laser beam welding enables high manufacturing precision and locally concentrated thermal input into the material at high processing speeds. However, the trend in the design of flow channels for bipolar plates shows an ever-growing tendency towards narrower channels (0.1 mm in the channel width)^6^. These dimensions are achievable with stamping processes and are challenging for the joining processes. With a high beam quality single mode fiber laser emitting in continuous mode at 1064 nm, welds with high aspect ratios can be realized. The weld seam width remains an issue and is usually ≥ 0.1 mm, allowing almost no positioning tolerances^7^.

The aim of this paper is to investigate the possibility of producing weld seams with a width of < 75 µm by means of laser beam welding. A newly developed QCW fiber laser with a wavelength of 532 nm and a high beam quality is used. Available lasers on the market emitting in the green range with powers suitable for material welding are so far disk lasers with significantly lower beam qualities (M^2^ ~ 25). By halving the wavelength from 1064 to 532 nm a theoretical reduction of the focus diameter by 50% is expected. The influence of the focus diameter on the energy input, the weld width and the weld shape are analyzed. For this purpose, process windows, in which defect-free overlap welding is possible at welding speeds of 500 mm/s and above, are identified. The system used is then evaluated in terms of its suitability for welding in narrow channels with a width of less than 75 µm.

## State of the art

### Laser beam welding

Laser beam welding belongs to the group of fusion welding. Here, a laser beam is used as the energy carrier, which is guided from the laser beam source onto the workpiece with the aid of flexible glass fibers and mirrors. A focusing lens is used to shape the laser beam according to the requirements of the manufacturing process. To position the laser beam on the workpiece and to enable path-shaped processing, a relative movement between the laser beam and the workpiece is required. This relative movement can be done by steering the laser beam by means of mirrors integrated in the beam path (in a galvanometer scanning head for example) or by moving the workpiece by means of an external device^8^. Lasers are generally used in two different operating modes. A distinction must be made between continuous wave mode and pulsed wave mode. In continuous wave mode (cw mode), the laser is operated continuously. In pulsed wave mode, the laser beam is interrupted at regular intervals to generate pulses^9^.

**Figure 1:**
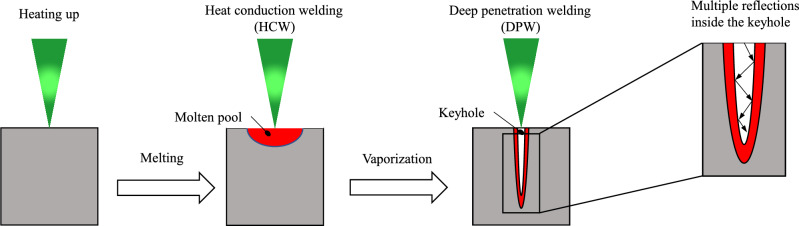
Heat conduction and deep penetration welding^11^.

Laser beam welding can be divided into two categories based on the outcome: heat conduction welding (HCW) and deep penetration welding (DPW) (see Fig. 1). The difference between these two lies in the energy input and the seam geometry. In heat conduction welding, the laser beam melts the surface of the material. The material is not heated above its vaporization temperature and the energy is only introduced into the material via thermal conduction. The weld geometry is lenticular with an aspect ratio of A ≈ 1 (ratio of weld penetration depth to weld width). During deep penetration welding, the vaporization temperature of the material is reached at the focal point of the laser beam. This causes a vapor capillary to form in the molten pool. In this vapor capillary, multiple reflections of the laser beam at the capillary walls occur, so that the radiation of the laser penetrates deeper into the workpiece. The resulting weld geometry is slender with a high aspect ratio (A ≥ 10)^10^.

### Influence of the wavelength

For laser material processing to be efficient, the energy from the laser radiation must be introduced into the material and converted into heat. The conversion of laser energy into process heat is referred to as energy coupling. With increasing energy coupling, the heat input into the material increases. Since the thermal energy introduced has an additional effect on the temperature-dependent optical material properties, the phase transitions caused, and the associated geometrical properties, this is referred to as interaction between the laser beam and the workpiece. The ratio of the coupled power to the power P incident on the workpiece is called the absorptance A, and is a measure of the available power P_abs_, which can be defined according to the following Eq. (1)^10^

1 $${\varvec{A}}=\boldsymbol{ }\frac{{{\varvec{P}}}_{{\varvec{a}}{\varvec{b}}{\varvec{s}}}}{{\varvec{P}}}$$

A: absorptance (–); P_abs_: absorbed power(W); P: incident power (W).

The remaining power is either reflected at the material surface or transmitted through the material.

2 $$1={\varvec{A}}+{\varvec{R}}+{\varvec{T}}$$

A: absorptance (–); R: reflectance (–); T: transmittance (–).

The reflectance R, the absorptance A and the transmittance T can be calculated from the Eq. (2), where each can take a value between 0 and 1^10^. In the case of welding metal foils with a thickness ≫ used wavelength, the transmittance T is negligible, and the formula is then expressed in terms of the reflectance and the absorptance with their addition equaling up to one.

**Figure 2:**
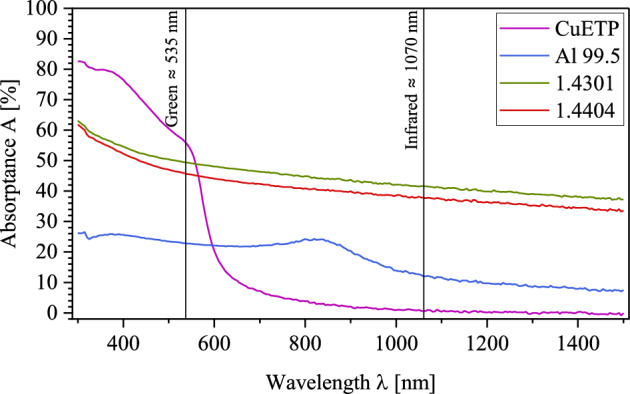
Absorptance of copper, aluminum, and stainless steel as a function of wavelength at room temperature.

The absorptance depends, among other things, on the material. Its value for stainless steel (1.4301 and 1.4404) increases at shorter wavelengths. Figure 2 gives an overview of the absorptance of different metals in relation to the wavelength of the laser beam source used at perpendicular beam incidence and room temperature. The coefficient for stainless steel is about 37% for an infrared laser beam source with a wavelength of λ =  ~ 1070 nm and 45% in the lower, green wavelength range λ =  ~ 535 nm. Therefore, the use of a laser beam source in the low, green wavelength range is suitable for material processing of steel.

### Spot diameter

In the field of laser beam welding, the beam quality and the focus ability of the laser beam source are of considerable importance. Thus, a higher beam quality would cause the focus diameter to fall below a critical minimal focus diameter d_0_ and consequentially to a higher welding depth under otherwise unchanged conditions. To enable a comparison of laser beam sources of the same power, the beam density—referred to as brilliance in laser technology—is used. The brilliance depends on the output power P_L_ of the laser as well as on the beam quality M^2^ and the wavelength λ and can be calculated using the Eq. (3)^12,13^

3 $${\varvec{B}}\boldsymbol{ }=\boldsymbol{ }\frac{{{\varvec{P}}}_{{\varvec{L}}}}{{{\varvec{\pi}}}^{2}\cdot {{\varvec{\omega}}}_{0}\cdot {{\varvec{\theta}}}_{0}}\boldsymbol{ }=\boldsymbol{ }\frac{{{\varvec{P}}}_{{\varvec{L}}}}{{({{\varvec{M}}}^{2}\cdot {\varvec{\uplambda}})}^{2}}$$

B: brilliance [W⁄(mm^2^∙sr)]; P_L_: output power (W); ω_0_: focal radius (mm); θ_0_: far field divergence angle (mrad); M^2^: beam quality (–); λ: wavelength (mm).

Decreasing brilliance causes an increase in the minimum achievable focal radius ω_0_, which must be considered when selecting the laser beam source. The beam quality M^2^ describes the deviation of the real laser beam from a theoretical ideal laser beam with a Gaussian beam distribution^13^. The propagation of a Gaussian beam is determined by the radius of the beam waist ω_0_ and by the wavelength λ. At a large distance from the beam waist, the increase of the beam radius is linear at the far field divergence angle θ_0_. Since ω_0_ can be arbitrarily adjusted with different lenses, the beam parameter product (BPP) is specified to characterize the laser beam. The beam parameter product is deduced from the beam quality M^2^ and the wavelength λ and expressed as follows in Eq. (4)^12^

4 $${\varvec{B}}{\varvec{P}}{\varvec{P}}\boldsymbol{ }=\boldsymbol{ }{{\varvec{M}}}^{2}\cdot \boldsymbol{ }\frac{{\varvec{\uplambda}}}{{\varvec{\pi}}}\boldsymbol{ }=\boldsymbol{ }{{\varvec{\omega}}}_{0}\boldsymbol{ }.\boldsymbol{ }{{\varvec{\theta}}}_{0}$$

BPP: beam parameter product (mm mrad); M^2^: beam quality (–); λ: wavelength (mm); ω_0_: beam waist radius (mm); θ_0_: far field divergence angle (mrad).

With the help of the beam parameter product, the theoretical focal diameter can be calculated according to^12^

5 $${{\varvec{d}}}_{0}=2\cdot {{\varvec{\omega}}}_{0}=2\cdot {\varvec{B}}{\varvec{P}}{\varvec{P}}\cdot \frac{{\varvec{f}}}{{{\varvec{\omega}}}_{{\varvec{R}}}}$$

d_0_: beam waist diameter (mm); ω_0_: beam waist radius (mm); BPP: beam parameter product (mm mrad); f: focal length of the focusing lens (mm); ω_R_: raw beam radius (mm).

In addition to the beam parameter product, the influencing characteristics of the spot diameter are the focal length of the focusing lens f and the raw beam radius ω_R_, which is available before the focusing lens. A reduction of the focus diameter can therefore only be achieved by reducing the focal length or by increasing the raw beam diameter^13^. Furthermore, reducing the used wavelength from 1070 to 535 nm leads to smaller foci.

### Humping

**Figure 3:**
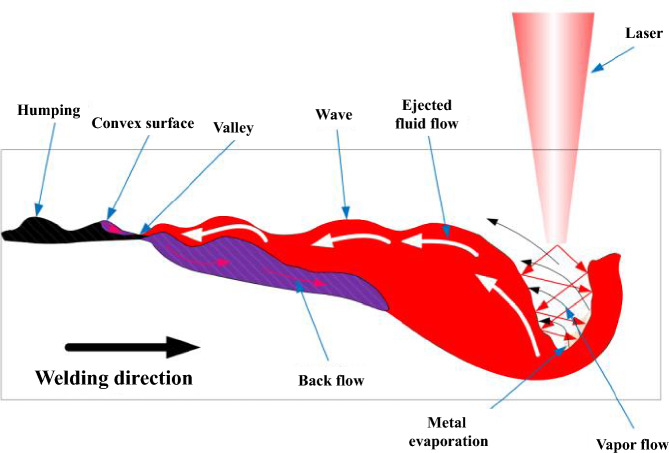
Formation of humping in a molten pool during welding^15^.

In high-speed welding, weld defects occur above a certain welding speed. While wave crests and troughs can initially be observed on the weld surface (pre-humping), drops (humps) form in the weld pool as the welding speed further increases. This effect is referred to as humping. Ai et al.^14,15^ explain the humping effect with the wave formation in the molten pool. During the welding process, the molten material is pushed backwards from the rear wall of the keyhole in gradually increasing waves against the feed direction. As can be seen in Fig. 3, the surface tension in the weld pool produces a convex surface. The narrowest point—the valley between the convex surface and the highest wave—solidifies at a higher cooling rate. This prevents backflow and periodic humps are herein formed^15^.

The main factors for the formation of humps are the inclination angle of the keyhole, a long and narrow melt pool, and colliding liquid flows in the melt pool^15,16^. The tilt angle of the keyhole increases as the welding speed increases, causing the vapor plume to further melt the metal at the back of the keyhole and lengthening the melt pool. A narrow weld pool causes a high backward vapor pressure, which causes an increase in the flow rate at which the melt is forced into the back of the molten pool. As a result, the height of the convex weld ejections increases inverse proportional to the width of the molten pool. The last factor to be mentioned is the collision of the backward-flowing molten pool with the backflowing, solidifying melt. The high momentum of the melt causes the formation of waves in the melt pool. This prevents backflow due to rapid solidification in the wave troughs and generates humps^15^. The limiting velocity above which humping occurs can be influenced by various process variables. Neumann et al. cite as such the material used, the focal length of the focusing lens used, the beam angle at which the laser beam impinges on the workpiece, the shielding gas used, and the laser power used^14^. Brodsky et al. show that a preheating of the material, for example using a ring laser, allows an increase of the maximum speed by 55% without humping effect. It is also shown that the humping distance, i.e., the distance between two humps, increases with increasing line energy^17^.

The line energy is calculated according to the Eq. (6)

6 $${\varvec{E}}=\frac{{\varvec{P}}}{{\varvec{v}}}$$

E: energy per unit length (J/mm); P: power (W); v: welding speed (mm/s)^10^.

## Experimental setup

### Laser beam source

In this paper, experiments are carried out with a single mode fiber laser beam source emitting in the visible green wavelength range (532 nm). Laser power and welding speed are varied and three different focusing lenses are used. Table 1 shows the technical data of the laser beam source.

**Table 1:** Specifications of the used laser beam source.

Laser parameters	min	typ	max
Wavelength $\lambda$ (nm)	530	532	534
Pulse repetition frequency $PRF$ (MHz)	120	180	210
Pulse duration $\tau$ (ns)	1.0	1.2	–
Average output power $P$ (W)	500	525	–
Peak output power ${P}_{max}$ (W)	–	2500	–
Beam quality ${M}^{2}$ (–)	1.1	1.2	–
Beam radius (mm)	–	0.6	–

The laser (Model: GLPN-500-R from the company IPG Photonics, Massachusetts USA) has a maximum average output power of about 500 W and emits in the visible green range with a wavelength of 532 nm. The Gaussian output beam has a beam quality of M^2^ = 1.2, achieving a beam parameter product (BPP) of 0.2 mm mrad. The system does not operate continuously as in continuous wave (CW) operation, but continuous wave operation is simulated by successive pulses with a pulse repetition rate of up to 210 MHz and a short pulse duration of 1.2 ns. This mode is called Quasi CW mode.

The laser can be operated in two modes: automatic current control mode (ACC mode, diode current is the control variable) and automatic power control mode (APC mode, output power is the control variable). A power measurement shows significant fluctuations in the output power. With the APC mode, a more constant output power can be generated in the low power range, but with this control mode the measured power does not reach the set power fast enough to be used for higher welding speeds. In ACC mode there is a deviation of less than 8% between measured power and target power in the range of 250–500 W, significant power losses are however measured in the power range below 250 W. In this mode, the laser does not emit any radiation if the power is set to 200 W and below.

### Setup and material configuration

**Figure 4:**
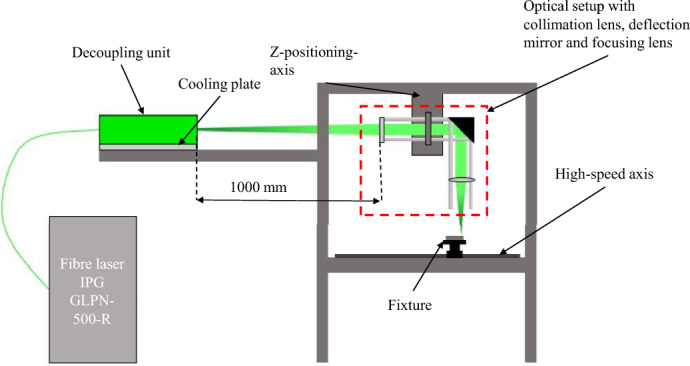
Schematic illustration of the welding setup.

This beam source decouples the laser beam via a separate decoupling unit in the form of a freely propagating laser beam with a (full) opening angle of 10 mrad, for which an individual, shield-free beam guidance must be realized. An existing experimental table with a finely adjustable z-axis and a perforated grid plate as a working plate serves as the basic framework for the setup. A specially developed software interface allows the welding parameters to be set and all machinery to be remotely controlled from a shielded control cabinet. Starting from the beam source, the laser beam is guided via a short fiber optic cable to a water-cooled decoupling unit, where it is decoupled. The output coupler unit is integrated into the beam path of the optical setup and aligned. The laser beam is focused on the workpiece surface, which is located on a high-speed axis with an integrated lifting table. The setup is shown simplified schematically in Fig. 4.

The divergent beam emerging from the decoupling unit must be parallelized. For this purpose, a collimating lens made of fused silica with a focal length of f = 1000 mm is inserted into the beam path at the corresponding propagation length, so that a collimated beam radius of ω_R_ = 5 mm is achieved. Subsequently, the collimated beam hits a deflection mirror, which is inclined by 45° and deflects it vertically downwards. For the laser beam welding process, focusing of the laser beam is necessary to melt the material of the workpiece. Focusing is done by means of a focusing lens made of fused silica, which is switched out three times to achieve different beam properties as shown in Table 2. The exact description of each lens from the providing company Thorlabs GmbH, Bergkirchen, Germany is LA4148-A-ML for the f50-lens, LA4380-A-ML for the f100-lens and LA4102-A-ML for the f200-lens.

**Table 2:** Lenses used with the calculated focus diameters and Rayleigh lengths based on the theoretical values from the laser datasheet.

Lens	f50-lens	f100-lens	f200-lens
Collimated beam diameter (mm)	10	10	10
Focal distance $f$ (mm)	50	100	200
Calculated focus diameter ${d}_{0}$ (µm)	4.06	8.12	16.24
Calculated Rayleigh length ${z}_{R}$ (µm)	20.3	81.2	324.8

The workpiece is mounted on an electromagnetic Linax Lxs highspeed axis from Jenny Science, Rain Switzerland. This can be used to weld on the workpiece surface at a welding speed of up to v = 2000 mm/s. Since the clamping jaws of the high-speed axis provide for lateral clamping, an attachment is designed and manufactured which allows the specimen to be clamped in such a way that a weld seam can be welded from above. The attachment consists of a plastic-printed T-piece and an aluminum plate onto which the sheet is pressed with two clamping plates. By screwing the clamping plates to the aluminum plate, thermal distortion of the sheets during the welding process is counteracted. An overview of the chemical composition of the three stainless steels considered in this investigation is shown in Table 3.

**Table 3:** Chemical composition of the materials (wt%)^18,19^.

	C	Si	Mn	P	S	Cr	Mo	Ni	N
1.4310 (AISI 301)	0.15	2.0	2.0	0.045	0.015	16.0–19.0	0.80	6.0–9.5	0.10
1.4404 (AISI 316L)	0.03	0.75	2.0	0.045	0.030	16.0–18.0	2.0–3.0	10.0–14.0	0.10
1.4301 (AISI 304)	0.07	0.75	2.0	0.045	0.030	17.5–19.5	–	8.0–10.5	0.1

**Figure 5:**
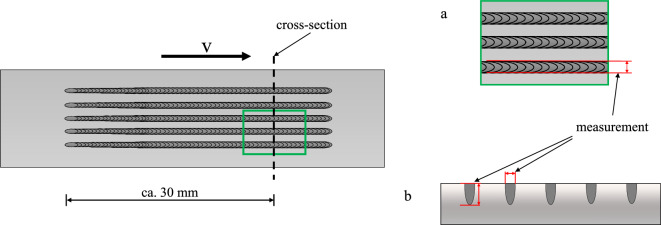
The measuring method for the welding width on the surface (a) and for the depth and width in the cross section (b).

The resulting weld seam width using this laser beam source and the setup is closely examined. The measuring method is shown in Fig. 5. The weld seam width is measured on microscopic images of the surface of the welded samples using a Keyence VHX 6000, Japan. This step is completed for all five welded lines before using a destructive grinding, polishing, and etching preparation to determine the weld seam width in the cross-section.

## Results and discussion

### Defining the process parameter window

Within the scope of this paper, bead on plate welds on stainless steel 1.4310 are carried out to evaluate the weld formation at small focal diameters (< 20 μm). Since the theoretically smallest focus diameter can be generated with the f50 focusing lens, an investigation of the welding parameters in the lower speed range up to v = 500 mm/s and a power up to P = 300 W are carried out with this lens. For this purpose, welds are performed on plates of stainless steel 1.4310 with a thickness of d = 500 µm. The aim is to analyze how the seam width of the weld seams changes and whether a weld penetration depth of 0.2 mm is achieved. This welding depth is relevant for the welding of two 0.1 mm thick foils in the production of bipolar plates. Subsequently, high welding speeds, up to v = 2000 mm/s and powers up to P = 500 W are investigated with the f100 and f200 focusing lens. An in-situ analysis is not possible in this case, so the welds are all examined ex-situ using microscopic images and metallographic preparation of the samples. Each power used is first welded at v = 2000 mm/s, which is then reduced by 200 mm/s in each parameter set in the following tests. The welding speed is reduced to its lowest value of v = 600 mm/s or when the weld overshoots (penetration depth ≥ sample thickness), corresponding to a weld penetration depth of  ≥ 0.5 mm. For a better overview, the parameter variations applied in these trials are listed in Table 4. The trials with the f50 lens are carried out in APC mode, since these are rather in the lower power range. For each parameter combination, five parallel welds are welded at 1 mm from each other on a 20 mm × 50 mm sample. The length of a weld seam is 40 mm.

**Table 4:** Overview of the parameter variations with each focusing lens.

Focusing lens	Theoretical focus diameter ${d}_{0}$ (µm)	Actual output power P (W)	Welding speed v (mm/s)
f50	4.06	100–300 (APC mode)	200–500
f100	8.12	200–500 (ACC mode)	400–2000
f200	16.24	200–500 (ACC mode)	600–2000

With the help of the above-mentioned trials, the process window is defined. Using data collected from cross-sections, the following findings can be stated:

- with the f50 (d_0_ = 4.06 µm), the goal of a penetration depth of  ≥ 0.2 mm is reached with a maximum welding speed of 300 mm/s and a 300 W output power. A maximum welding speed of 200 mm/s with an output power of 200 W also achieves the required goal. With an output power of 100 W, the criterium is not met. Due to the welding depth with this lens being significantly lower than expected, no higher welding speeds are examined in this configuration (f50 and APC mode).
- with the f100 (d_0_ = 8.12 µm), the window parameter is larger: with the lowest output power of 200 W and a welding speed of 600 mm/s, a weld seam depth of  ≥ 0.2 mm is reached. With the maximum set output power of 500 W, this criterium is met at the maximum welding speed of 1800 mm/s.
- with the f200 (d_0_ = 16.24 µm), the highest welding speed amongst all the trials (2000 mm/s) and an output power of 300 W lead to the required welding depth.

**Figure 6:**
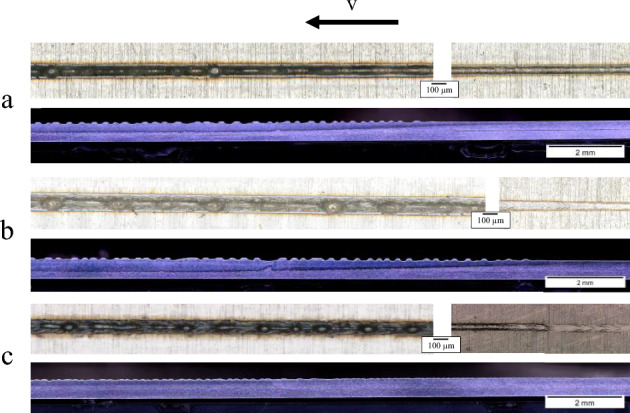
Surface image of the beginning and middle parts of the weld seam and longitudinal section with (**a**) f100, 300 W, 1000 mm/s, (**b**) f100, 400 W, 1000 mm/s and (**c**) f200, 500 W, 1800mm/s.

A further insight into the kind of weld seams created with this laser can be acquired by longitudinal sections. Three parameter sets are chosen from the previous trials. The microscopic images of the weld seam surface in the beginning range, mid-range and the corresponding longitudinal sections are presented in Fig. 6. The three parameter sets are:

- f100, P = 300 W and v = 1000 mm/s
- f100, P = 400 W and v = 1000 mm/s
- f200, P = 500 W and v = 1800 mm/s

The welding direction is in this case from right to left. As to be expected with these higher welding speeds, the weld seams show clear signs of the humping phenomena, which usually occurs at welding speeds ≥ 500 mm/s. The samples are chemically etched so the weld seam depth is visible due to darker shading. Notably, the welding depth shows an almost linear increase for a significant starting length of the weld seam (about 8 mm), with the laser beam not coupling until after a few millimeters from the start of the radiation. This can be justified by looking at the step response of the laser itself. In ACC mode, this step response has a duration of 5.5 ms^20^. Heussen investigated the response of the laser and the power output in detail in^20^. At a welding speed of 1000 mm/s for a and b, the laser beam travels 5.5 mm (or almost 14% of the total length) over the stainless-steel workpiece before reaching the set output power. Measured at the left side in the image, after the depth has stabilized, the value is then 0.25 mm and 0.33 mm for a and b and 0.194 mm for c. In the case of the parameter set c, the distance travelled is even higher at 9.9 mm. That is just under 25% of the total length of the weld seam. The correspondent weld seam widths on the top surface are ~ 0.1 mm, ~ 0.115 mm and ~ 0.09 mm. Another important finding in this case, is the appearance of a significant decrease in the weld seam depth with parameter sets a and b, right before the maximum value is reached. This effect is heightened by the humping formation as well.

### Evaluating the weld seam width and depth

**Figure 7:**
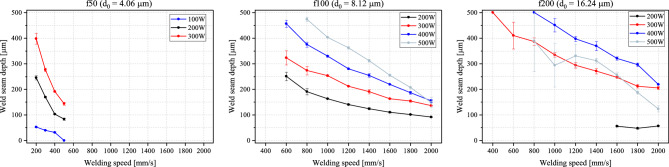
Weld seam depth plotted against the welding speed with the different lenses.

The weld seam width and depth are major characteristics and are usually the used criteria to evaluate the welding process itself. For bipolar plate applications, these are even more crucial. A fluctuation in the penetration depth can lead to gaps between the half-plates, thus the joined plate cannot fulfill the criterium of being hermetically sealed. Maximizing the active area available within a bipolar plate is advantageous. One technique to achieve this is by scaling down the gas flow channels’ width and maximizing their number. For this purpose, thinner weld seams are strived for. The used laser beam source in this paper has a high beam quality (one of the highest available on the market today) and a high brilliance, making it a good tool for such an analysis. In this section, an overview of the results reached with the previously mentioned trials is presented. Microscopic imaging and cross sections of the welded seams are used for this evaluation. In Fig. 7, the diagrams show how the weld seam depth changes in accordance with the welding speed at different output powers and using the three different focusing lenses.

An overall trend is that with an increasing welding speed, the weld penetration depth decreases. This is to be expected and well known from previous literature. With the f50, only three data points fulfill the requirement of a welding depth ≥ 0.2 mm: at output powers of 200 W and 300 W and welding speeds of 200 mm/s and 300 mm/s. These welding speeds are comparatively low and not suitable for the application in bipolar plate welding. Promising results with the highest reproducibility—due to restricted standard deviation—are achieved with the f100 lens. Opposite to what can be expected, with the larger spot diameter with this lens (about 8 µm) in comparison to the previous lens (about 4 µm), the values for the welding depth are higher at a higher welding speed. Most of the data points are above the 200 µm line. With the f200 lens, the results show a large fluctuation, particularly with the 500 W curve. A counter-intuitive finding from evaluating the results is that with the increasing spot diameter, thus an increasing surface area with a wider energy distribution, the welding depth increases. This is particularly noted for the 300 W and 400 W powers. A similar trend is observed in the work of^21^, where it is shown, that below 200 µm beam waist, the focus ability and penetration depth are mainly influenced by the BPP and the divergence angle.

**Figure 8:**
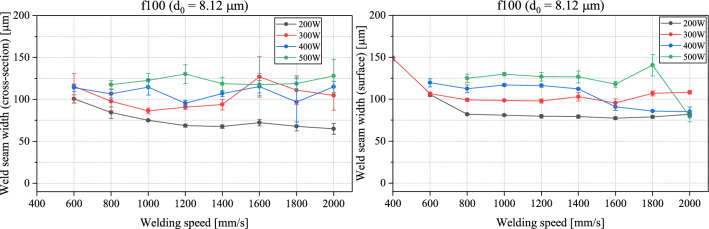
Weld seam width in the cross-section (left) and on the surface (right) plotted against the welding speed with different set powers for f100.

**Figure 9:**
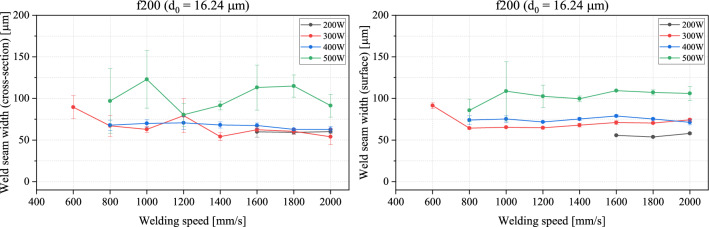
Weld seam width in the cross-section (left) and on the surface (right) plotted against the welding speed with different set powers for f200.

The results are plotted in the following graphs in Figs. 8 and 9. Each data point represents the averaged-out measurement from five weld seams and the standard deviation shows the degree of fluctuation. A first observation shows that the welding speed starting 800 mm/s has for the most part no significant influence on the achieved welding width, but rather that the weld seam width is mainly affected by the output power of the laser. This is mainly true for the weld seam width on the surface of the samples for both f100 and f200 with the only exception at higher welding speeds (1600 mm/s) with f100, where the 300 W curve rises above the 400 W curve. In the cross-section, the margin of error is too large to reach a definitive conclusion from the obtained results in this range. The weld seam width seems to not be distinctively higher or lower with a particular power. The deviations between the results on the surface and in the cross-section can be justified mainly with human error and due to welding defects caused by humping effects appearing on the surface. With the f100, the weld seam width stays below 150 µm for almost all the measured data. With the f200, almost all data points remain below the 125 µm line, with only three data points having a standard deviation that crosses it [500 W at 800 mm/s and 1000 mm/s (cross section) and 500 W at 1000 mm/s (surface)]. Overall, it can be deduced that thinner weld seams are achieved with the f200. With the f200 lens, the smallest divergence angle is achieved. The BPP is kept unchanged for all the used lenses since the same laser is being used. A possible explanation for the occurrences observed in this work, is that with the f200 and due to the small divergence angle, the laser radiation is further reflected downwards in the keyhole causing a narrower and deeper weld seam. Whereas with the f100 and f50, the laser radiation—due to the larger divergence angles—is absorbed and reflected at a lower penetration depth, causing the welds to be larger in width and shallower, confirming the results from^21^.

In another evaluative aspect, certain trials are closely examined. The results here show a large fluctuation, particularly with the higher power of 500 W. This can be attributed to factors such as process instabilities and human error. While the investigation aims at targeting the same distance in the metallographic preparation of the samples, this is not a guaranteed outcome. The measurements are also carried out using microscopic images, which can lead to some faulty readings, particularly the occurrence of humping and defining of the limits of the weld seam causing the reading to slightly differ from sample to sample. Here, the criterium for the weld seam depth is to be on average above 0.2 mm and below 0.3 mm. At the same time, the weld seam width must be at a minimum for both the surface and the cross-section. In this case, the parameter sets listed in Table 5 deliver promising results. This shows that with the f200, reaching weld seam widths lower than 75 µm (0.075 mm) is possible.

**Table 5:** The minimal weld seam widths achieved with the f100 and f200 lenses.

Lens used	Laser power (W)	Welding speed (mm/s)	Weld seam width (cross-section) (µm)	Weld seam width (surface) (µm)
100	300	1000	86	99
100	300	1200	91	98
200	300	1200	79.4	65
200	300	1400	54.2	68
200	300	1600	59	71
200	300	1800	60.4	70
200	300	2000	54	74
200	400	1800	62.8	75
200	400	2000	62.75	71

### Humping analysis

Humping is a phenomenon which occurs when welding at higher welding speeds, usually above 500 mm/s. The molten pool is pushed upwards due to dynamic flow characteristics around the keyhole. This is recognizable by the appearance of so-called humps and destabilizes the weld penetration depth. This negatively impacts the joining of two bipolar plate halves. Many strategies are being developed to further understand the formation of these humps and to try and push the welding speed limit upwards. In the scope of this paper, no strategies are implemented to prevent humping. This chapter aims to give an overview of the samples where this phenomenon takes place and to try and deduce a relevance with the increasing speed used to carry out the welding trials.

No signs of humping are observed with all the f50 trials. From the rest of the welds, only four in total have no humping:

- f100: a power of 200 W at welding speeds of 400 mm/s and 600 mm/s, and a power of 300 W at a welding speed of 400 mm/s
- f200: a power of 300 W at a welding speed of 300 mm/s

**Figure 10:**
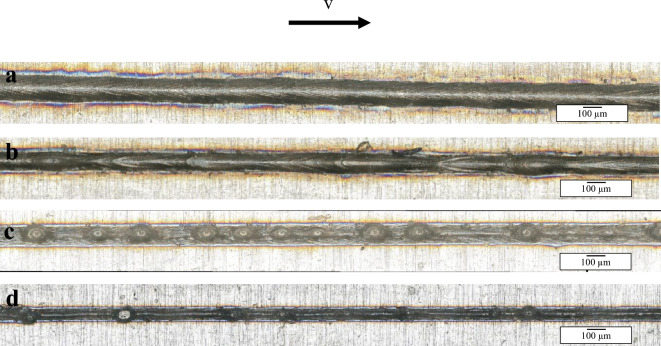
Top view of welded samples with (**a**) f100, 300 W, 400 mm/s, (**b**) f100, 300 W, 600 mm/s, (**c**) f100, 300 W, 1000 mm/s and (**d**) f200, 300 W, 1400 mm/s.

In Fig. 10 images of four parameter sets are illustrated. These show, that with increasing welding speed (starting 600 mm/s), some pre-humping signs start to show (b). This is a symptom of the molten stream starting to collect in the middle, however no clear humps are formed. In Fig. 10 a, the welding speed is 400 mm/s, and the weld seam surface is smooth with no signs of humping. Notably, in the remaining two images (c and d), the weld seam width is decreasing as the welding speed increases, while humps form along the middle of the welded line. The distance between each hump is increased with f200 and a higher welding speed.

## Summary

This work investigates the possibility of producing weld seams with a width of < 75 µm by means of laser beam welding. A newly developed green fiber with a wavelength of 532 nm is used. The influence of the focus diameter on the energy input, the weld width and the weld shape are evaluated. For this purpose, process windows are identified in which the weld seam width and depth are suitable for the mentioned application. The weld penetration depth needs to be higher than 200 µm, while remaining below 300 µm. The weld seam width needs to be minimized. Using such a system in terms of its suitability for welding in narrow channels, such as the gas flow channels found on bipolar plates for PEM fuel cells, is then evaluated.

First, welding trials with three different focusing lenses f50, f100 and f200 are carried out. These allow theoretical spot diameters of about 2 µm, 4 µm and 8 µm. In a first attempt, the f50 is found to not deliver expected results and is excluded from further evaluation. The laser offers two kinds of control modes: ACC and APC. ACC mode is more suitable for output powers starting at 250 W and has a faster response time. Through microscopic imaging and longitudinal sections, the response time of the laser is found to have a significant effect on the welding result, since the sample travels 5.5 mm before the desired output power is reached. Promising results are achieved with f100 and f200, particularly using powers of 300 W and 400 W.

Furthermore, a significant finding is that the welding speed above 800 mm/s and up to 2000 mm/s has a reduced effect on the result with regards to the weld seam width, which highly depends on the set power. The lowest weld seam width is achieved with the f200 lens. The results here show mainly four parameter sets which are possibly suitable for welding in thin channels with a width < 75 µm. These are:

- f200, 300 W, 1400 mm/s
- f200, 300 W, 1600 mm/s
- f200, 300 W, 1800 mm/s
- and f200, 400 W, 2000 mm/s

Finally, with regards to defects, the humping defect is observed with most of the trials. The reason for this is the higher welding speeds used (starting at 600 mm/s), which are the typical boundary for the appearance of this phenomenon.

## Data Availability

The authors declare that the data supporting the findings of this study are available within the paper. Supplementary information files and data can be made available upon request.
